# Numb-PRRL promotes TGF-β1- and EGF-induced epithelial-to-mesenchymal transition in pancreatic cancer

**DOI:** 10.1038/s41419-022-04609-y

**Published:** 2022-02-23

**Authors:** Weiwei Sheng, Jingtong Tang, Rongxian Cao, Xiaoyang Shi, Yuteng Ma, Ming Dong

**Affiliations:** 1 Department of Gastrointestinal Surgery, The First Hospital, China Medical University, 110001 Shenyang, Liaoning China; 2 Department of Hernia and Abdominal Wall Surgery, Chaoyang Hospital, 100043 Beijing, China

**Keywords:** Tumour-suppressor proteins, Cell invasion

## Abstract

Isoform-specific functions of Numb in the development of cancers, especially in the initiation of epithelial-to-mesenchymal transition (EMT) remains controversial. We study the specific function of Numb-PRRL isoform in activated EMT of pancreatic ductal adenocarcinoma (PC), which is distinguished from our previous studies that only focused on the total Numb protein. Numb-PRRL isoform was specifically overexpressed and silenced in PC cells combining with TGF-β1 and EGF stimulus. We systematically explored the potential effect of Numb-PRRL in the activated EMT of PC in vitro and in vivo. The total Numb protein was overexpressed in the normal pancreatic duct and well-differentiated PC by IHC. However, Numb-PRRS isoform but not Numb-PRRL showed dominant expression in PC tissues. Numb-PRRL overexpression promoted TGF-β1-induced EMT in PANC-1 and Miapaca-2 cells. TGF-β1-induced EMT-like cell morphology, cell invasion, and migration were enhanced in Numb-PRRL overexpressing groups following the increase of N-cadherin, Vimentin, Smad2/3, Snail1, Snail2, and cleaved-Notch1 and the decrease of E-cadherin. Numb-PRRL overexpression activated TGFβ1-Smad2/3-Snail1 signaling was significantly reversed by the Notch1 inhibitor RO4929097. Conversely, Numb-PRRL silencing inhibited EGF-induced EMT in AsPC-1 and BxPC-3 cells following the activation of EGFR-ERK/MAPK signaling via phosphorylating EGFR at tyrosine 1045. In vivo, Numb-PRRL overexpression or silencing promoted or inhibited subcutaneous tumor size and distant liver metastases via regulating EMT and Snail signaling, respectively. Numb-PRRL promotes TGF-β1- and EGF-induced EMT in PC by regulating TGF-β1-Smad2/3-Snail and EGF-induced EGFR-ERK/MAPK signaling.

## Introduction

Derived from the epithelium of the pancreatic duct, pancreatic ductal adenocarcinoma (PC) is an aggressive malignant disease with a 10% rate of 5-year overall survival [1] and with the second-highest mortality in the next few years [2]. A hallmark of PC is its remarkable local invasion and distant metastasis, and frequent chemoresistance [3], which is strongly accelerated by epithelial–mesenchymal transition (EMT) during tumor development.

Once an EMT program is activated, cancer cells lose epithelial characteristics (intercellular contacts and polarity) and gain mesenchymal properties (migratory and invasive capacities) following the unbalanced expression of epithelial (E-cad) and mesenchymal biomarkers (Fibronectin, Vimentin, and N-cadherin), which finally contributes to cancer invasion and metastasis [4]. EMT is identified as a critical cancer hallmark that increasing studies have been lent to genetic, molecular, and biochemical mechanisms in revealing the relationship between the EMT process and cancer metastasis. Notably, TGF-β1 (transforming growth factor beta1) and EGF (epidermal growth factor), plays a significant role in driving EMT program based on our previous and current studies [3, 5–7].

Numb was the first molecule discovered as a critical cell fate determinant in Drosophila [8]. It displayed complex and multiple functions including the control of asymmetric cell division and cell fate choice, endocytosis, cell adhesion and migration, ubiquitination of specific substrates, and a variety of signaling pathways (Notch, Hedgehog, MDM2-p53, and WNT signaling) [8–10]. Accumulated evidence showed that Numb acts as a tumor-suppressive role in medulloblastoma [10], breast cancer [11], non-small cell lung cancer (NSCLC) [12], ovarian cancer [13], and colorectal cancer [14]. On the other hand, overexpression of Numb was found in hepatocellular carcinoma [15], astrocytomas [16], and cervical squamous carcinoma cells [17]. The opposite role of Numb in cancers reflects the different functions and distribution of its multiple isoforms. Indeed, alternative splicing of Numb transcripts produces two major isoforms that differ in the length of their proline-rich region (PRR), due to inclusion (PRRL) or exclusion (PRRS). In murine embryonic carcinoma cells, Numb-PRRS (molecular weight 65/66 kD) and Numb-PRRL (molecular weight 71/72 kD) were involved in differentiation and proliferation, respectively [18]. This different role of each isoform could explain the contrasting functions of Numb in different cancer types. Our previous studies showed that Numb inhibited cell invasion, migration, and chemoresistance in PC cells via preventing p53 ubiquitin-dependent degradation [19]. Additionally, Musashi2 promotes the development and progression of PC by down-regulating Numb [20]. However, we just focused on the function of total Numb protein previously. In the current study, we intend to investigate the specific function of Numb-PRRL isoform in the initiation of EMT of PC, which supplied a novel and much more precious gene target for PC intervention.

## Materials and methods

### Tissue samples and PC cell lines

Our current research was authorized by the academic committee from the first hospital of China Medical University. PC Patients with radical surgical resection enrolled in this study accepted the specimen consent. As described previously [18, 19], 79 cases of radical resected paraffin-embedded PC and additional 65 cases of the paired pancreas were picked up from surgical treatment patients from 2010 to 2020 which were definitely diagnosed as ductal adenocarcinoma. Patients with endocrine carcinoma, acinar cell carcinoma, and invasive intraductal papillary mucinous carcinoma were excluded from this study. 16 PC tissue samples were collected for WB and PCR assays, respectively. Four cell lines (AsPC-1, BxPC-3, PANC-1, and Miapaca-2) were purchased from the National collection of authenticated cell culture in Shanghai with recommended growth media containing 10% fetal calf serum (Hyclone, Logan, UT, USA) without mycoplasma contamination.

### Immunohistochemistry (IHC) assays

IHC was performed as described previously [19, 20]. Briefly, tissue sections were deparaffinized and dehydrated, incubated with H_2_O_2_, subjected to high-pressure repair, and blocked with BSA. anti-Numb (Abcam, Cambridge, UK, dilution: 1:400), Snail1 (Proteintech, Chicago, IL, dilution: 1:200), Snail2 (Proteintech, dilution: 1:200), E-cad (Abcam, 1:1000), N-cad (Proteintech, dilution: 1:200), Vimentin (Proteintech, dilution: 1:500), and Ki67 (Proteintech, dilution: 1:500) overnight. Slices were covered with the secondary antibody, detected with DAB, co-stained with hematoxylin, and evaluated by two professional pathologists for the staining scores.

### Immunoblot assays

Whole-cell lysates were prepared from PC specimens and cell lines. Cells were pretreated with 10 ng/ml of TGF-β1 (Peprotech, RockyHill, New Jersey, USA) plus Notch signaling inhibitor RO4929097 (10 μM, Selleckchem, USA) for 24 h to detect the activity of TGF-β1-Smad2/3-Snail signaling. All samples were loaded onto 10% SDS-polyacrylamide gels, transferred to PVDF membranes (Millipore Corp, Bedford, MA, USA), and incubated with primary Numb (Abcam,1:1000), ZEB-1 (Proteintech, 1:1000), Fibronectin (Proteintech, 1:1000), E-cadherin (E-cad, Abcam, dilution: 1:1000), N-cadherin (N-cad, Proteintech, 1:1000), Vimentin (Proteintech, dilution: 1:1000), Smad2/3 (Cell Signaling Technology, Beverly, USA, dilution: 1:500), p-ERK (Cell Signaling Technology, dilution: 1:1000), Snail1 (Proteintech, 1:500), Snail2 (Proteintech, 1:500), cleaved-Notch1 (Cell Signaling Technology, dilution: 1:1000), phosphorylating EGFR at tyrosine 1045 (p-EGFR1045, Cell Signaling Technology, dilution: 1:1000) and GAPDH (Proteintech, 1:3000) overnight at 4 °C. Membranes were incubated with horseradish peroxidase-conjugated secondary antibodies (Proteintech) for 2 h at room temperature. Immunoreactive protein bands were visualized with an ECL detection kit (Thermol Biotech Inc, USA). Each experiment was repeated three times.

### qRT-PCR

As described previously [19, 20], mRNA from PC tissues and cell lines was analyzed in a Light Cycler 2.0 with the Light Cycler kit (Takara Bio, Japan). The primers were as follows: Numb-PRRL, 5’-CTTCCAAGCTAATGGCACTG-3’ (sense) and 5’-CTCTTAGACACCTCTTCTAACCA-3’ (antisense); Numb-PRRS, 5’-CAATCTCCTACCTTCCAAGGG-3’ (sense) and 5’-CGGACGCTCTTAGACACCTC-3’ (antisense). The quality of the PCR products was monitored with post-PCR melt-curve analysis. The expression level of these target genes was calculated by the −ΔΔCt method.

### siRNA, Crispr/cas9, and lentivirus vector-mediated Numb-PRRL overexpression

Two specific interference sequences for Numb-PRRL were: sense: 5′CCUUCCAUGUGCUUGCUAATT3′; antisense: 5′UUAGCAAGCACAUGGAAGGTT3′; sense: 5′GCAAUGCCUGUGCGUGAAATT3′; antisense: 5′UUUCACGCACAGGCAUUGCTT3′. All of them were synthesized from GenePharma company (GenePharma Co, Ltd, Shanghai, China). siRNA transfections were mixed with Oligofectamine 3000 (Invitrogen, Carlsbad, CA, USA) under the manufacturer. Using the same interference sequence, Crispr/cas9 system-mediated Numb-PRRL silencing (sgNumb-PRRL) and scramble (sgRNA) stable cell lines were constructed (Genechem, Shanghai, China). Lentivirus vector-mediated Numb-PRRL overexpression (Numb-PRRL-GFP) and scramble (GFP) were purchased from Genechem (Shanghai, China). Because the full-length mRNA sequence of Numb-PRRL isoform is the longest one among all the transcripts, only Numb-PRRL isoform could be specifically silenced that did not affect PRRS isoform expression. Thus, PANC-1 and Miapaca-2 cells with low Numb-PRRL expression were used for constructing Numb-PRRL overexpressing stable cell lines following puromycin treatment, whereas AsPC-1 and BxPC-3 cells with high Numb-PRRL expression were available for Numb-PRRL silencing.

### EMT construction

PANC-1 and Miapaca-2 cells cultured with 1% FBS growth media were simultaneously treated with 10 ng/ml TGF-β1 (Peprotech) daily for 3 days, while AsPC-1 and BxPC-3 cells were treated with 50 ng/ml EGF (Peprotech) daily for 3–4 days. 1%BSA (Sigma, USA) was used as a control. EMT-like cell morphology, cell invasion and migration, and the change of epithelial and mesenchymal biomarkers were used to reveal EMT formation in vitro.

### Cell invasion and migration assays

According to our previous study [19, 20], Numb-PPRL overexpressing or silencing PC cell lines were implanted into membrane inserts (BD Biosciences, USA) covered with 10%matrigel in 24 well plates with free serum medium combing with EGF and TGF-β1 stimulus. Medium combining 10% FBS was incubated at the bottom as the stimulus. The migrated cells under the bottom of the inserts were fixed and co-stained with crystal violet (Sigma). The final cell migrated numbers were calculated in at least five random fields/each well. The migration assay was conducted in a similar way without matrigel. This experiment was repeated at least three times.

### In vivo xenograft model

All animal work was performed and approved under the Institutional Animal Care of China Medical University according to the national guidelines. The 8-week-old nude mice (BALB/c, female) were randomly assigned to each group and the investigator was blinded to the group allocation. Based on our previous study, all the cell lines in the current study are reliable to construct a subcutaneous tumor model. However, Miapaca-2 and AsPC-1 cells are much easier to construct liver metastasis than PANC-1 and BxPC-3 cells [7, 21, 22]. Liver metastasis is a common feature of pancreatic cancer. After spleen injection, the tumor cells go through splenic vein via spleen vein, subsequently return to the portal vein, and finally transfer to the liver. Because we find no primary splenic tumor at the endpoint of observation. Thus, the spleen was preserved after spleen injection to avoid serious surgical trauma to the nude mice. This protocol is the common method for the liver metastasis model in pancreatic cancer [23]. Numb-PRRL-GFP/GFP transfected PANC-1 cells and sgNumb-PRRL/sgRNA transfected BxPC-3 cells were randomly injected into axillae of nude mice to construct subcutaneous tumor formation. Mice were kept for 20d before sample harvest. Tumor volumes were calculated by the following formula: length × width × height × 0.5 in mm. Besides, Numb-PRRL-GFP/GFP transfected Miapaca-2 cells and sgNumb-PRRL/sgRNA transfected AsPC-1 cells were randomly injected into the spleen of nude mice to construct a distant liver metastasis model. Briefly, Cells (1 × 10^7^/ml) mixed with pre-cold PBS (150 μl) were slowly injected into the lower-middle part of the spleen. A cotton swab was pressed toward the injection site to avoid bleeding and leakage. The samples that were harvested 30 days later were assessed according to the number of liver metastases. All samples were next fixed for hematoxylin and eosin (HE), and IHC staining.

### Statistical analysis

Based on the SPSS software 21.0 (Chicago, IL, USA), the differences in WB assay, cell migration and invasion assays, subcutaneous tumor volumes, and the number of liver metastases were compared through Student’s *t* test. Non-parametric and Spearman correlation tests were analyzed for IHC assays in vivo and human PC samples. The association of Numb expression with clinicopathological data was analyzed by Chi-squared test and Spearman correlation test. *P* < 0.05 or *P* < 0.01 was considered significant.

## Results

### Total Numb protein and Numb-PRRL expression in PC tissues

**Fig. 1:**
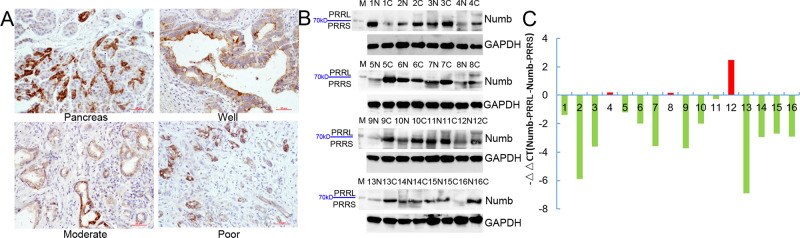
Total Numb protein and Numb-PRRL expression in PC tissues. **A** Total Numb protein expression in normal pancreas, well-to-poor differentiated PC tissues. **B**, **C** Numb-PRRL and PRRS protein (**B**) and mRNA (**C**) expression in 16 cases of PC and paired normal pancreas.

We first detected total Numb protein expression in 79 cases of PC and 65 cases of normal pancreas. IHC showed that the positive expression of total Numb protein was 45.5% (36/79) in PC and 38.4% (25/65) in the normal pancreatic duct (Fig. 1A). Indeed, Numb was only overexpressed in well-differentiated PC (70%, 21/30), but weakly expressed in moderated (34.3%, 11/32) and poorly (23.5%, 4/17) differentiated PC tissues, respectively (Table 1). Spearman correlation analysis further verified that Numb expression was closely associated with tumor differentiation (*r* = −0.381, *P* = 0.002) (Table 1). Thus, we next detected the expression of Numb-PRRL (71/72 kD) and PRRS (65/66 kD) isoforms based on their different molecular weight. WB showed that Numb-PRRL expression was much lower in 16 PC samples than that of Numb-PRRS expression (*P* < 0.001; Fig. 1B). Using specific primes of these two major isoforms, qRT-PCR further showed that only 3 samples had high Numb-PRRL mRNA expression (PRRL 3/16 vs PRRS 13/16) (Fig. 1C). Based on the above results, Numb-PRRS, but not Numb-PRRL, governed the expression and function of total Numb protein in PC, which acts as a tumor-suppressive role in PC in accordant with our previous studies [18, 19].

**Table 1:** The relationship between Numb and tumor differentiation.

Parameters		Numb		*r*	*P*
		Negative	Positive		
Tumor differentiation	High	9	21	−0.381	0.002
	Moderate	21	11		
	Low	13	4		
Total		43	36		

### Construct of Numb-PRRL overexpressing and silencing cell lines

**Fig. 2:**
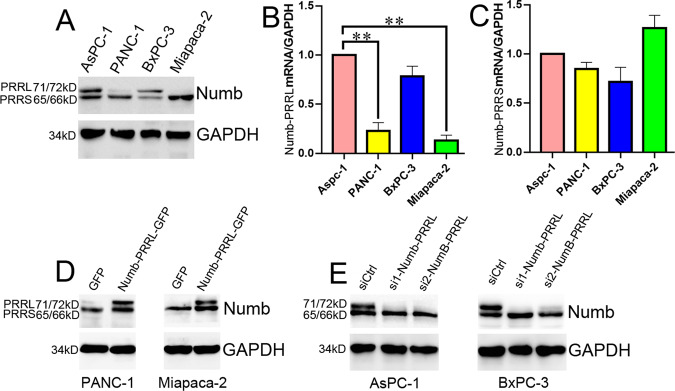
Construct of Numb-PRRL overexpressing and silencing cell lines. **A** Numb-PRRL and PRRS protein (**A**) and mRNA (**B**, **C**) expression in 4 PC cell lines. **D** Construct of Numb-PRRL overexpressing PANC-1 and Miapaca-2 cells. **E** Construct of Numb-PRRL silencing AsPC-1 and BxPC-3 cells. Bars indicate ±S.E. *P* < 0.05; **P < 0.01 compared with control.

However, Numb-PRRL was prevalently expressed in several PC cell lines. WB and qRT-PCR showed that Numb-PRRL mRNA and protein (71/72kD) expression was much higher in AsPC-1 and BxPC-3 cells, but was lower in PANC-1 and Miapaca-2 cells (Fig. 2A, B), while the mRNA and protein (65/66kD) expression of Numb-PRRS showed no significant difference (Fig. 2A, C). Therefore, PANC-1 and Miapaca-2 cells was used for constructing Numb-PRRL overexpressing stable cell lines via lentiviral transfection, whereas AsPC-1 and BxPC-3 cells were available for Numb-PRRL silencing by siRNA. WB showed that Numb-PRRL was overexpressed in PANC-1 and Miapaca-2 cells (Numb-PRRL-GFP group) compared with that in the GFP group (Fig. 2D), whereas it was significantly silenced in AsPC-1 and BxPC-3 cells compared with that in siCtrl groups (Fig. 2E).

### Numb-PRRL promoted TGF-β1- and EGF-induced EMT like cell morphology in vitro

**Fig. 3:**
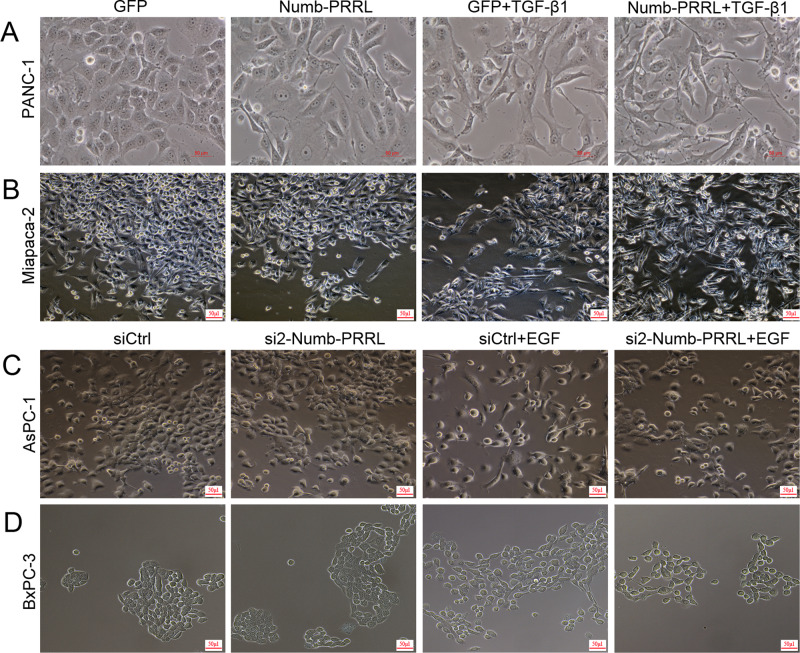
Numb-PRRL promoted TGF-β1- and EGF-induced EMT-like cell morphology in vitro. **A**, **B** Numb-PRRL overexpression promoted TGF-β1-induced EMT-like cell morphology in PANC-1 (**A**) and Miapaca-2 (**B**) cells. **C**, **D** Numb-PRRL silencing inhibited EGF-induced EMT-like cell morphology in AsPC-1 (**C**) and BxPC-3 (**D**) cells.

The transition of EMT-like cell morphology (mesenchymal-like phenotype) is the first step, once the EMT program is activated [4]. Our previous studies showed that TGF-β1 and EGF showed different sensitivity in stimulating EMT dependent on specific PC cell types [5, 7, 22]. Briefly, TGF-β1 had a sensitive effect in PANC-1 and Miapaca-2 cells, whereas EGF showed a better function in AsPC-1 and BxPC-3 cells [5, 7, 22]. In detail, under TGF-β1 treatment, most PANC-1 (49–54%) and Miapaca-2 (41–46%) cells lost their epithelial characteristics and presented a spindle-shaped and fibroblast-like morphology (Fig. 3A, B). Moreover, Numb-PRRL overexpression enhanced TGF-β1-induced function in PANC-1 (69–73%) and Miapaca-2 (58–64%) cells following with much obvious spindle-shaped and fibroblast-like morphology. Conversely, Numb-PRRL silencing inhibited EGF-induced EMT like cell morphology in AsPC-1 (71–78% in siCtrl vs 38–43% in si2-Numb-PRRL) and BxPC-3 (56–63% in siCtrl vs 33–39% in si2-Numb-PRRL) cells (Fig. 3C, D). Therefore, Numb-PRRL promoted TGF-β1- and EGF-induced EMT-like cell morphology in vitro.

### Numb-PRRL promoted TGF-β1- and EGF-induced cell invasion and migration in vitro

**Fig. 4:**
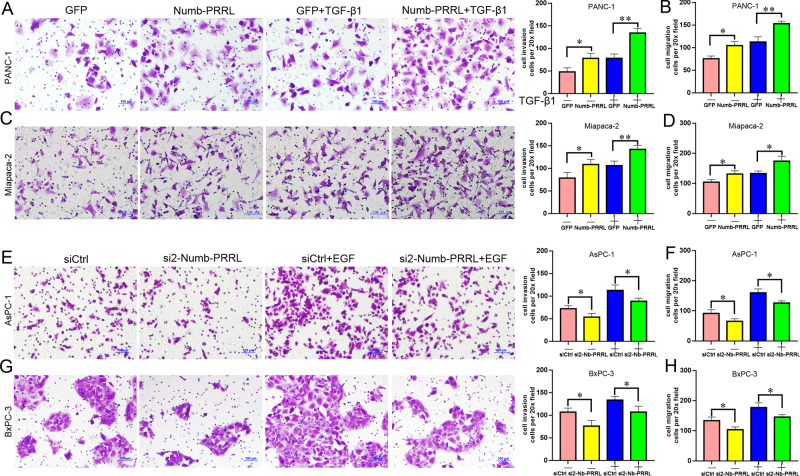
Numb-PRRL promotedTGF-β1- and EGF-induced cell invasion and migration in vitro. Numb-PRRL overexpression promoted TGF-β1- induced cell mobility in PANC-1 (**A**, **B**) and Miapaca-2 (**C**, **D**) cells. Numb-PRRL silencing inhibited EGF-induced cell mobility in AsPC-1 (**E**, **F**) and BxPC-3 (**G**, **H**) cells. Bars indicate ± S.E., *P < 0.05, **P < 0.01 compared with control.

Aggressive invasion is the key hall marker of EMT [3]. TGF-β1 induced cell invasion and migration in PANC-1 (Fig. 4A, B) and Miapaca-2 cells (Fig. 4C, D), which was significantly enhanced by the overexpression of Numb-PRRL. Upon TGF-β1, cell invasion and migration were significantly increased in Numb-PRRL-GFP groups compared with the corresponding GFP groups. Namely, the disparity of cell invasion and migration in the GFP groups with or without TGF-β1 treatment was less significant than that in the Numb-PRRL-GFP groups. Conversely, EGF induced cell invasion and migration in AsPC-1 (Fig. 4E, F) and Miapaca-2 cells (Fig. 4G, H). However, Numb-PRRL silencing reversed EGF-induced cell mobility in vitro. Therefore, Numb-PRRL promoted TGF-β1- and EGF-induced cell invasion and migration in vitro.

### Numb-PRRL stimulated TGF-β1-induced Smad2/3-Snail and EGF-induced ERK/MAPK signaling in vitro

**Fig. 5:**
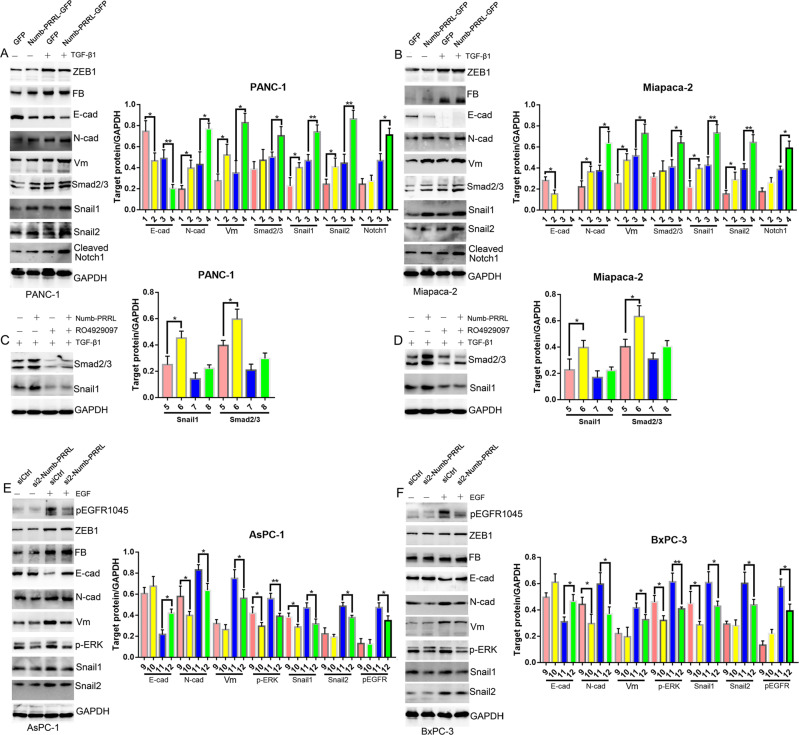
Numb-PRRL stimulating TGF-β1-induced Smad2/3-Snail and EGF-induced EGFR-ERK/MAPK signaling in vitro. **A**, **B** The EMT biomarkers, ZEB-1, Smad2/3, Snail1, Snail2, and cleaved-Notch1 expression in Numb-PRRL-GFP and GFP transfected PANC-1 (**A**) and Miapaca-2 (**B**) cells with or without TGF-β1 treatment. **C**, **D** The protein expression of Snail1 and Smad2/3 in Numb-PRRL-GFP and GFP transfected PANC-1 (**C**) and Miapaca-2 (**D**) cells with or without TGF-β1 and RO4929097 treatment. **E**, **F** The EMT biomarkers, p-EGFR1045, ZEB-1, p-ERK, Snail1, and Snail2 expression in si2-Numb-PRRL and siCtrl transfected AsPC-1 (**C**) and BxPC-3 (**D**) cells. 1. GFP group; 2. Numb-PRRL-GFP group; 3. GFP group plus TGF-β1; 4. Numb-PRRL-GFP group plus TGF-β1. 5. GFP group plus TGF-β1; 6. Numb-PRRL-GFP group plus TGF-β1; 7. GFP group plus TGF-β1 and RO4929097; 8. Numb-PRRL-GFP group plus TGF-β1 and R04929097, 9. siCtrl group; 10. si2-Numb-PRRL group; 11. siCtrl group plus EGF; 12. si2-Numb-PRRL group plus EGF group. Bars indicate ±S.E. *P < 0.05; **P < 0.01 compared with control.

We next investigated the potential mechanism based on the above results. Firstly, Numb-PRRL overexpression alone partially downregulated E-cad and upregulated N-cad and Vimentin expression in PANC-1 and Miapaca-2 cells (Fig. 5A, B). Meanwhile, Snail1 and Snail2, as the key transcript factor targeting E-cad, were also simultaneously activated (Fig. 5A, B). However, the protein level of Smad2/3, ZEB1, and Fibronectin was unchanged. Upon TGF-β1, the downregulation of E-cad and upregulation of N-cad and Vimentin were significantly enhanced in Numb-PRRL-GFP groups in comparison with the GFP group (Fig. 5A, B). Moreover, Numb-PRRL overexpression enhanced TGF-β1-induced Smad2/3, Snail1, and Snail2 expression in comparison with the GFP group. To our surprise, Numb-PRRL overexpression had no effect in cleaved-Notch1 expression but promoted cleaved-Notch1 protein level in TGFβ1-induced PANC-1 and Miapaca-2 cells. Meanwhile, the activation of Numb-PRRL overexpression on TGFβ1-induced Smad2/3 and Snail1 was significantly reversed by Notch1 inhibitor RO4929097 (Fig. 5C, D). Thus, Numb-PRRL and Notch signaling cooperatively promote TGFβ1-induced EMT in vitro.

EGF, as another critical EMT and ERK/MAPK signaling stimulus, not only induced p-EGFR1045, also triggers the decrease of E-cad and the increase of N-cad, Vimentin, p-ERK, Snail1, and Snali2 expression in AsPC-1 and BxPC-3 cells, respectively (Fig. 5E, F). Though Numb-PRRL silencing only partially downregulated N-cad and p-ERK expression, its silencing inhibited EGF-induced change of EMT and EGFR-ERK/MAPK signaling. In brief, EGF induced the decrease of E-cad and the increase of p-EGFR1045, N-cad, Vimentin, p-ERK, Snail1, and Snali2 expression were significantly reversed by Numb-PRRL silencing (Fig. 5E, F). Thus, Numb-PRRL silencing inhibited EGFR-ERK/MAPK signaling in EGF-induced EMT in vitro.

### Numb-PRRL overexpression promoted subcutaneous tumors formation and distant liver metastasis in vivo via regulating EMT and Snail1/Snail2 signaling

**Fig. 6:**
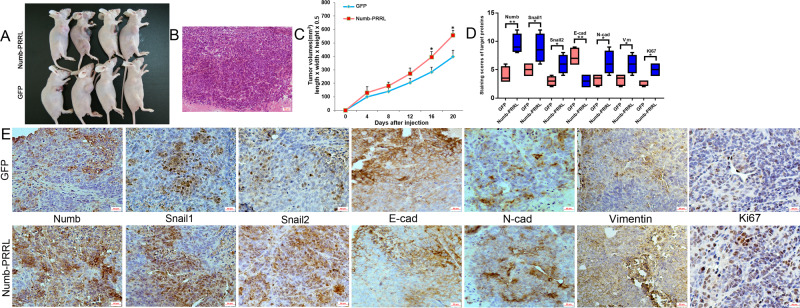
Numb-PRRL overexpression promoted subcutaneous tumors formation in vivo. **A** Tumor volumes in the Numb-PRRL and GFP groups implanted with PANC-1 cells. **B** HE staining of harvested tumor. **C** Tumor growth curve in Numb-PRRL and GFP groups. **D** The statistic data of IHC assays. **E** The different expression levels of Numb, Snail1, Snail2, E-cad, N-cad, Vimentin, and Ki67 in the Numb-PRRL and GFP groups by IHC. Bars indicate ±S.E. *P < 0.05; **P < 0.01 compared with control.

PANC-1 and Miapaca-2 cells were used to construct subcutaneous tumor and liver metastasis model, respectively. Tumor volumes in Numb-PRRL-GFP group were much larger than that of corresponding GFP groups in a time-dependent manner (Fig. 6A, C). The primary tumors were diagnosed by HE staining (Fig. 6B). Meanwhile, IHC verified that Numb, N-cad, Vimentin, Snail1, Snail2, and Ki67 were upregulated but E-cad was downregulated in Numb-PRRL-GFP groups in comparison with GFP groups (Fig. 6D, E), which was consistent with the results in vitro.

**Fig. 7:**
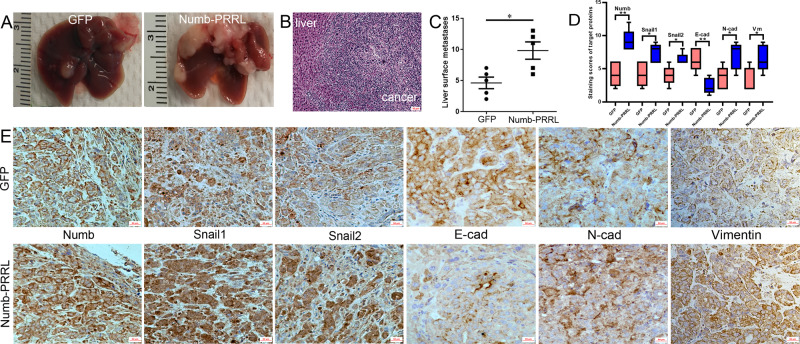
Numb-PRRL overexpression promoted distant liver metastasis in vivo. **A** Liver metastases in the Numb-PRRL and GFP groups implanted with Miapaca-2 cells. **B** HE staining of harvested tumor. **C** The statistic data involving the number of liver metastasis in Numb-PRRL and GFP groups. **D** The statistic data of IHC assays. **E** The different expression levels of Numb, Snail1, Snail2, E-cad, N-cad, and Vimentin in Numb-PRRL and GFP groups by IHC. Bars indicate ±S.E. *P < 0.05; **P < 0.01 compared with control.

The average number of liver metastases in the Numb-PRRL-GFP group was much higher than that of the GFP group (Fig. 7A, C). The distant liver metastases were diagnosed by HE staining (Fig. 7B). Similarly, IHC verified that Numb, N-cad, Vimentin, Snail1, and Snail2 expression was upregulated but E-cad was downregulated in Numb-PRRL-GFP groups compared with that in GFP groups (Fig. 7D, E), which was consistent with the results in vitro.

**Fig. 8:**
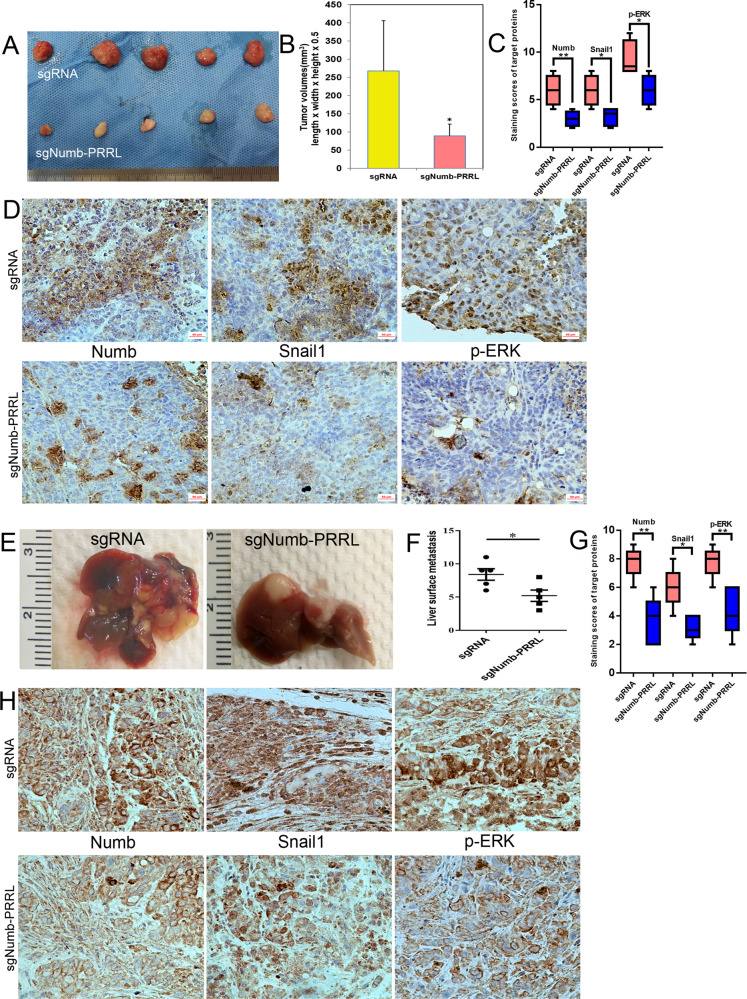
Numb-PRRL silencing inhibited subcutaneous tumor size and distant liver metastasis in vivo. **A**, **B** Tumor volumes in sgNumb-PRRL and sgRNA groups implanted with BxPC-3 cells. **C** The statistic data of IHC assays. **D** The different expression of Numb, Snail1, and p-ERK in sgNumb-PRRL and sgRNA groups by IHC in subcutaneous tumor tissues. **E** Liver metastases in sgNumb-PRRL and sgRNA groups implanted with AsPC-1 cells. **F** The statistic data involving the number of liver metastasis in these two groups. **G** The statistic data of IHC assays. **H** The different expression of Numb, Snail1, and p-ERK in sgNumb-PRRL and sgRNA groups by IHC in liver metastasis tissues. Bars indicate ±S.E. *P < 0.05; **P < 0.01 compared with control. Bars indicate ±S.E. *P < 0.05; **P < 0.01 compared with control.

Numb-PRRL silencing in BxPC-3 inhibited subcutaneous tumor size in vivo (Fig. 8). Tumor volumes in the sgNumb-PRRL group were smaller than that of the corresponding sgRNA group (Fig. 8A, B). IHC verified that Numb, Snail1, and p-ERK were downregulated in the sgNumb-PRRL group in comparison with the sgRNA group (Fig. 8C, D). Similarly, Numb-PRRL silencing in AsPC-1 cells inhibited distant liver metastases in vivo following the downregulation of Snail1 and p-ERK expression (Fig. 8E–H).

## Discussion

The distinguished roles of Numb in various cancers are largely dependent on the different distribution of its multiple isoforms. Interestingly, the same isoforms of Numb also play a contrasting function in different cancers. For example, Numb-PRRL promotes proliferation, migration, invasion and colony formation of hepatocellular carcinoma [15], but prevents EMT in esophageal squamous cell carcinoma cells [24]. In the current study, Numb-PRRL promotes TGF-β1- and EGF-induced EMT in PC in vitro and in vivo via regulating TGF-β1-Smad2/3-Snail and EGF-induced EGFR-ERK/MAPK signaling, which has not been reported yet.

Our previous studies showed a tumor-suppressive role of total Numb protein in the development of PC, which indicated that Numb-PRRS but not PRRL isoform dominated the function of a total Numb in PC. Thus, we detected the protein and mRNA expression of these two major isoforms in PC first. As we predicted, both protein and mRNA expression of Numb-PRRS in PC tissues were much higher than that of PRRL. Meanwhile, Numb-PRRS overexpression was associated with tumor high differentiation, indicating its pro-differentiation and tumor-suppressive role in the progression of PC based on our current and previous studies [19, 20, 25]. Interestingly, Numb-PRRL was overexpressed in hepatocellular carcinoma [14], medulloblastoma [10, 26] and invasive urothelial carcinoma of the bladder [27], but exhibited low expression in breast [11] and lung cancers [12]. Therefore, the unbalanced distribution of Numb isoforms contributes to the distinguished role of total Numb protein in cancers.

However, Numb-PRRL was prevalently expressed in several PC cell lines. Regarding Numb-PRRL as the longest transcript based on the NCBI database, only Numb-PRRL isoform could be specifically silenced that didn’t affect PRRS isoform expression. Thus, Numb-PRRL isoform was specifically overexpression and silencing in vitro. We first found that Numb-PRRL overexpression promoted TGF-β1-induced EMT in vitro. TGF-β1 induced EMT-like cell morphology, cell invasion, and migration, and the change of EMT biomarkers was enhanced by Numb-PRRL overexpression. Meanwhile, Numb-PRRL overexpression promoted TGF-β1-induced Smad2/3, Snail1, and Snail2 in vitro independent of ZEB1. TGF-β1 serves an important role in regulating cell growth, proliferation, differentiation, extracellular matrix synthesis, and the development of EMT [28, 29]. TGF-β1-Smad2/3-Snail signaling is the critical molecular pathway involving the development of EMT in several cancers, including lung cancer [30], breast cancer [31], liver [32], and PC [5]. TGF-β1 activates the receptor complex leads to the activation of Smad2 and Smad3 through direct C-terminal phosphorylation [33]. Activated Smad2/3 complexes cooperated with Smad4 to regulate target genes, including Snail1, Snail2, ZEB1, and TWIST1, all of which were central to EMT. TGF-β1 promotes EMT-dependent fibrosis via TGF-β1/Smad/Snail signaling [34], and induces sustained upregulation of Snail1 and Snail2 through Smad pathways in human corneal epithelial cells [35]. Recently, Notch signaling has been reported to promote TGF-β1-induced EMT of retinal fibrosis cells and contribute to fibrosis in vivo, both of which can be inhibited by Notch inhibitors [36, 37]. The cooperation of TGF-β with Notch signaling promotes EMT in different malignancies, such as ovarian cancer [38] and squamous cell carcinoma [39]. Consistent with previous studies, cooperation of TGF-β1 and Notch signaling promotes EMT in PC cells in the current study. However, to our surprise, Numb-PRRL overexpression enhanced cleaved-Notch1 expression in TGFβ1-induced PC cells. Numb-PRRL and Notch signaling cooperatively promote TGFβ1-induced EMT in vitro. In previous studies, total Numb protein, as a critical Notch signaling inhibitor, promotes Notch1 receptor ubiquitination and degradation of the Notch1 intracellular domain (NICD1) [40]. Total Numb negatively regulates the EMT of triple-negative breast cancer by antagonizing Notch signaling [41]. Conversely, Numb protein stabilizes the NICD1 by regulating the ubiquitin–proteasome machinery. [42]. In lung cancer, Numb-PRRS inhibits Notch receptor (NICD) activation, whereas the Numb-PRRL promotes NICD activation [43]. Taken together, Numb trends to regulate Notch signaling in its isoform-dependent manner. Numb-PRRL and Notch signaling cooperatively promote TGFβ1-induced EMT PC cells. The corresponding mechanism would be investigated in our future study.

EGF-induced EGFR-ERK/MAPK signaling plays a significant role in PC EMT based on our previous studies [3, 5, 7]. Calreticulin promotes EGF-induced EMT in PC via Integrin/EGFR-ERK/MAPK signaling pathway [3]. Musashi2 promotes EGF-induced EMT in PC via ZEB1-ERK/MAPK signaling [7]. In the current study, Numb-PRRL silencing inhibited EGF-induced EMT in vitro via EGFR-ERK/MAPK signaling. Numb-PRRL silencing inhibited EGF-induced p-EGFR1045, p-ERK, Snail1, and Snail2 independent of ZEB1. EGF also induces EMT through the Snail signaling pathway in breast cancer cells [44]. In addition, a synergistic effect between EGF and TGF-β1 was found in inducing EMT-related oncogenic properties of intestinal epithelial cells [45]. EGF promotes TGF-β1-induced EMT in HK-2 cells through a synergistic effect on Snail [46]. Thus, we inferred close crosstalk of Numb-PRRL in regulating TGF-β1 and EGF-induced EMT in PC with the final target of Snail signaling.

Finally, Numb-PRRL overexpression promoted subcutaneous tumors formation and distant liver metastasis in vivo along with the activation of EMT and Snali1/2 signaling. Conversely, Numb-PRRL silencing inhibited subcutaneous tumors formation and distant liver metastasis in vivo following the downregulation of Snali1 and ERK/MAPK signaling.

In conclusion, for the first time, we demonstrate Numb-PRRL promotes TGF-β1- and EGF-induced EMT in PC by activating TGF-β1-Smad2/3-Snail and EGFR-ERK/MAPK signaling in vitro and in vivo. Meanwhile, Numb-PRRL and Notch signaling cooperatively promote TGFβ1-induced EMT, which supplies a novel direct and new sight for PC treatment.

## Supplementary information

Original Data File

aj-checklist

## Data Availability

The data sets used and/or analyzed during the current study are available from the corresponding author on reasonable request.
