# Supplementary figures and images for: Numb-PRRL promotes TGF-β1- and EGF-induced epithelial-to-mesenchymal transition in pancreatic cancer
Source: Cell Death Dis. 2022 Feb 23;13(2):173. doi: 10.1038/s41419-022-04609-y (PMC8866481; doi:10.1038/s41419-022-04609-y)

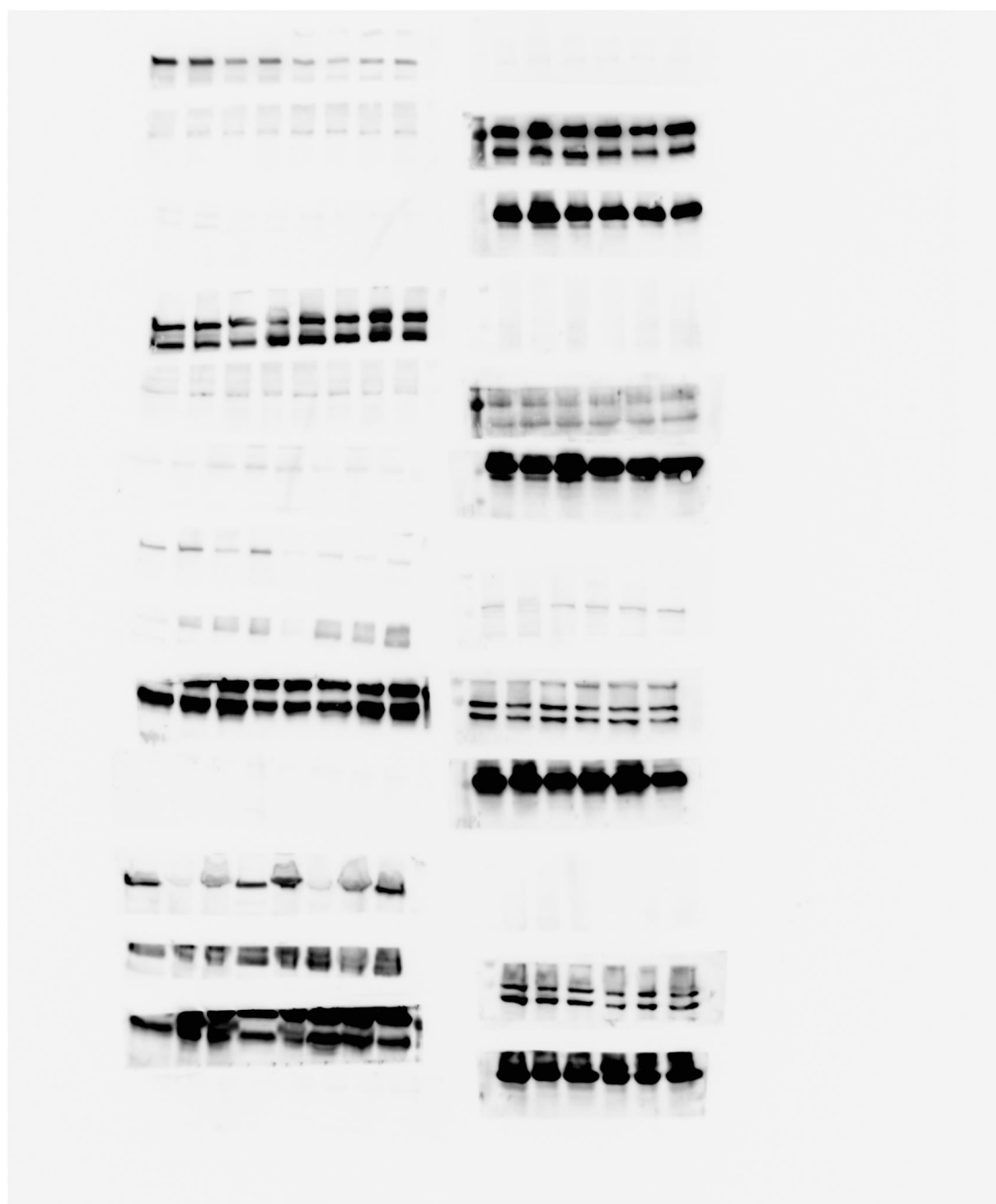

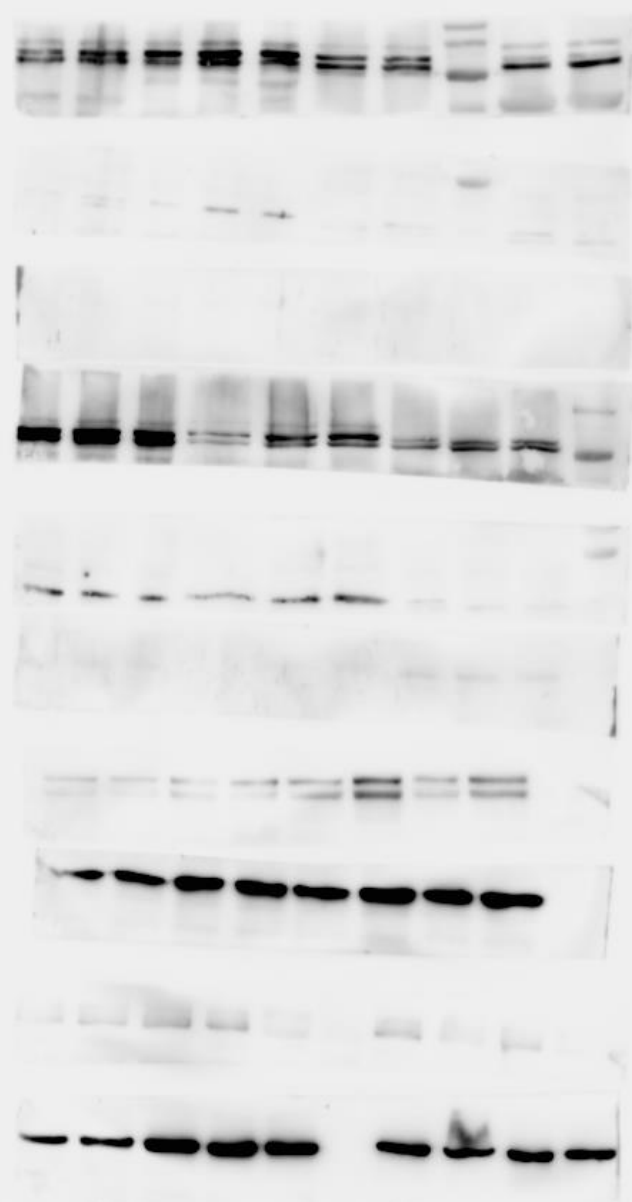

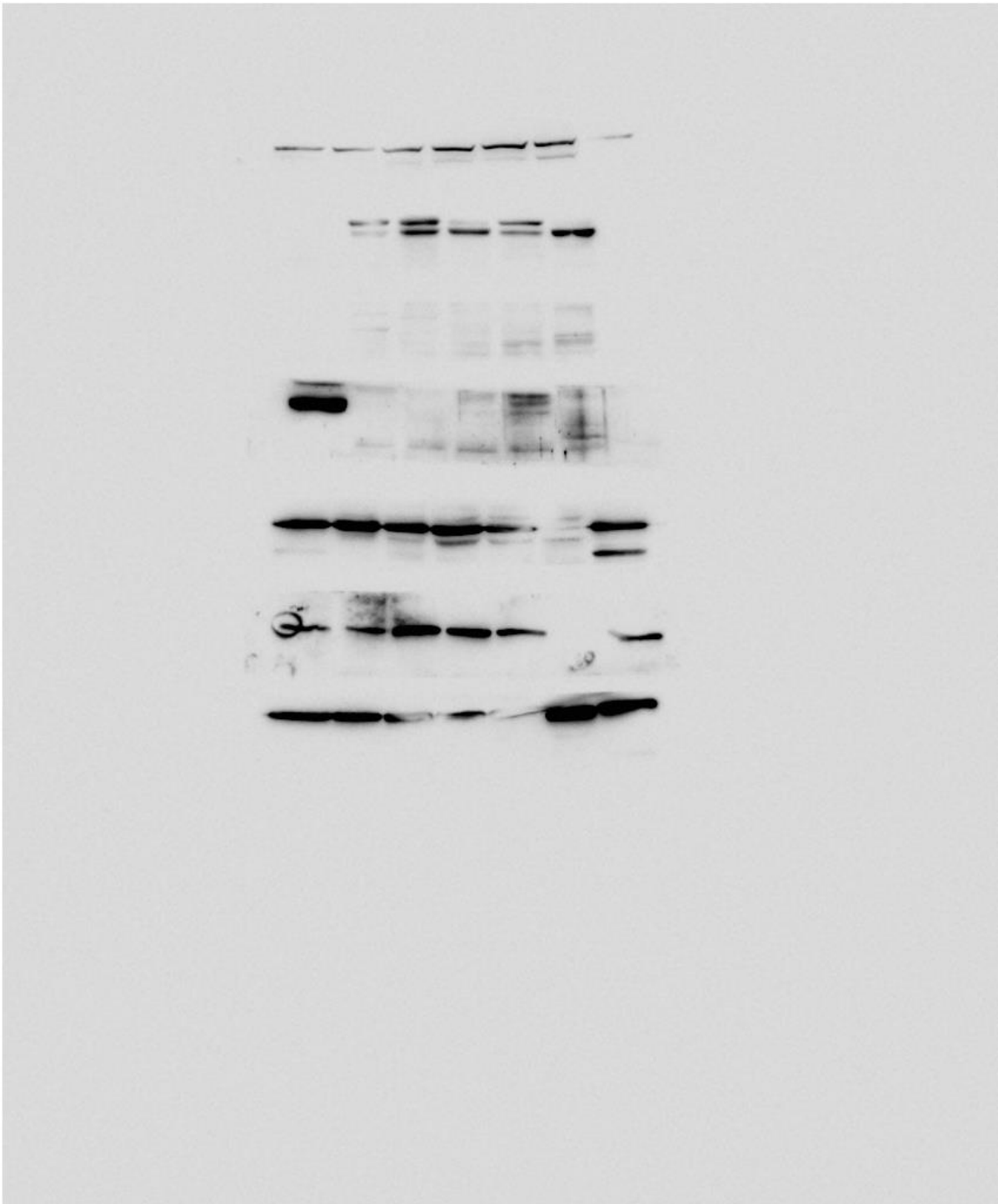

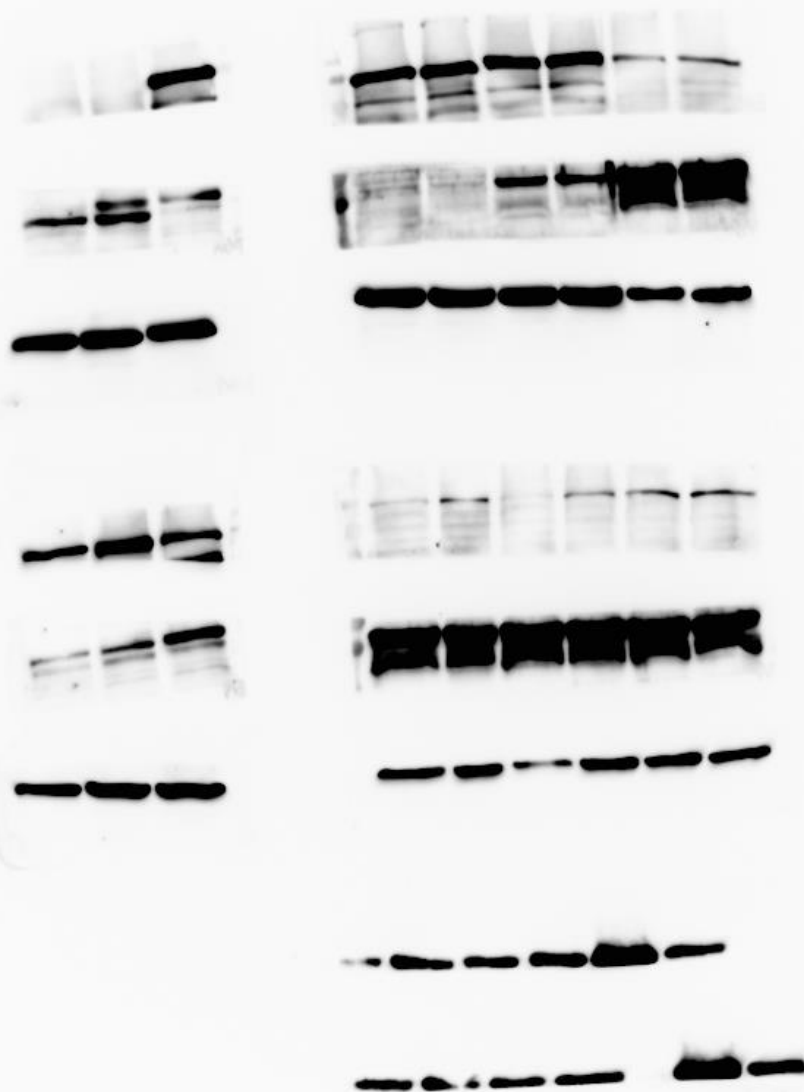

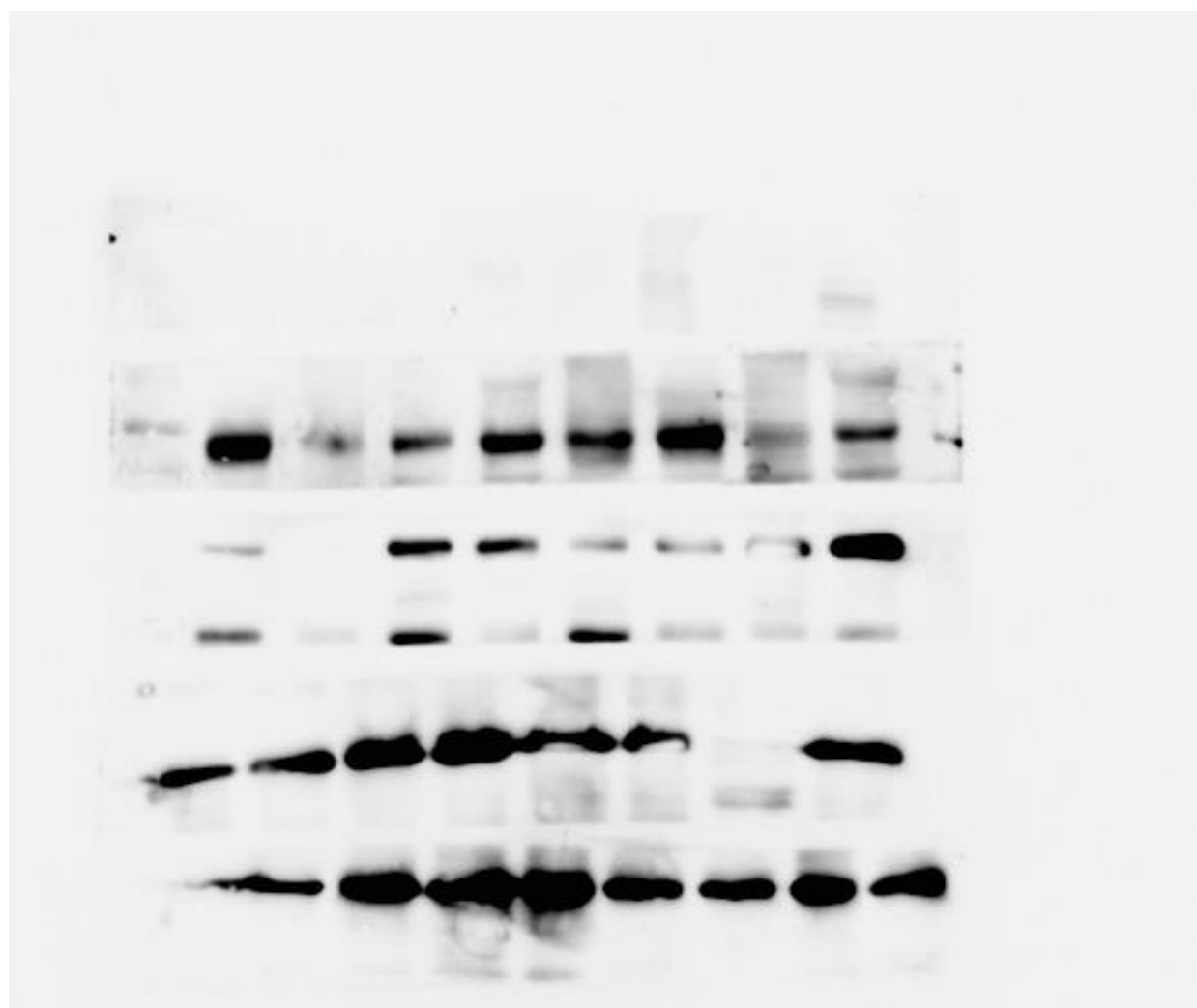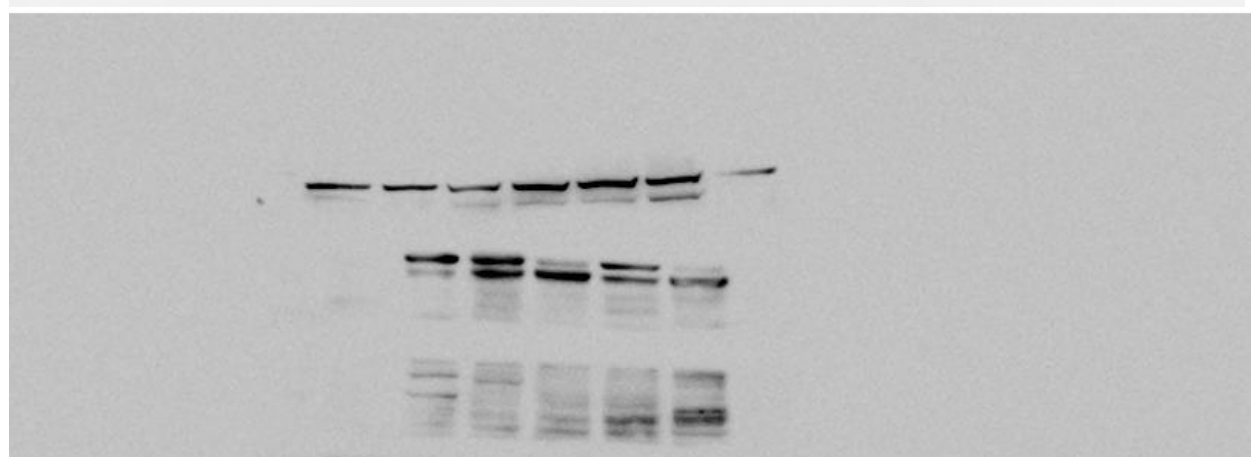

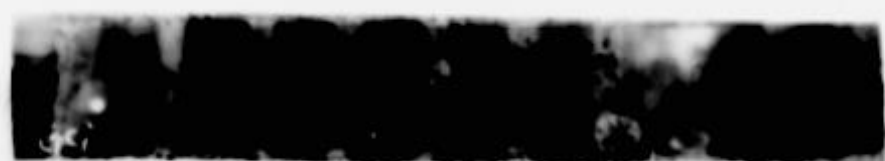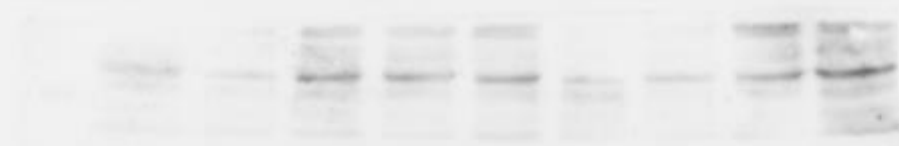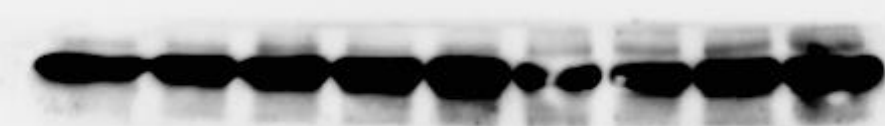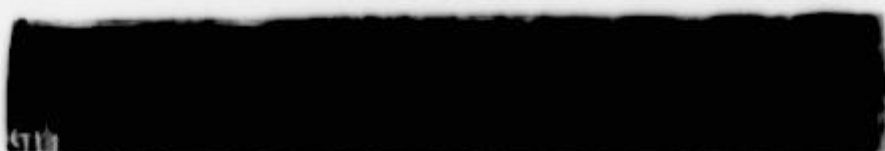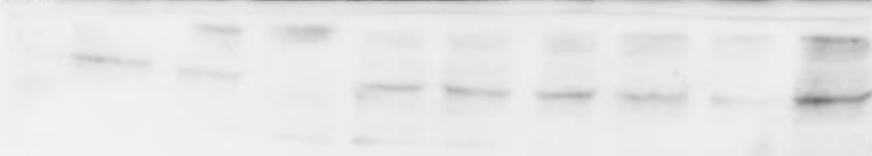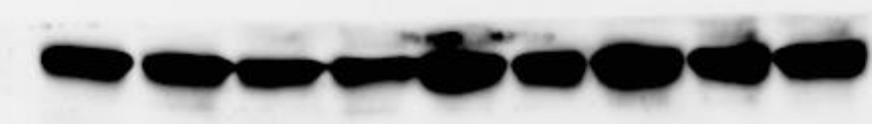

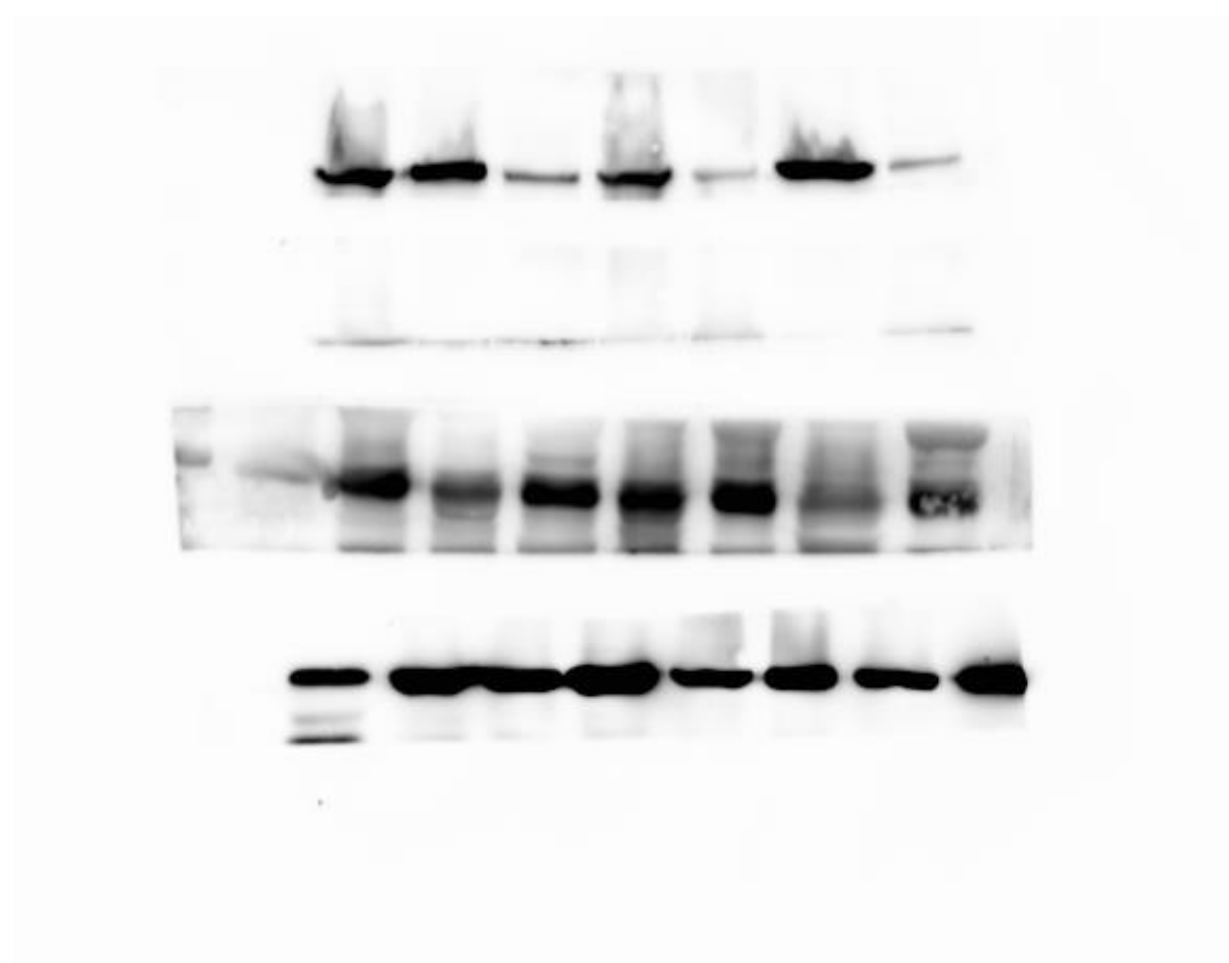

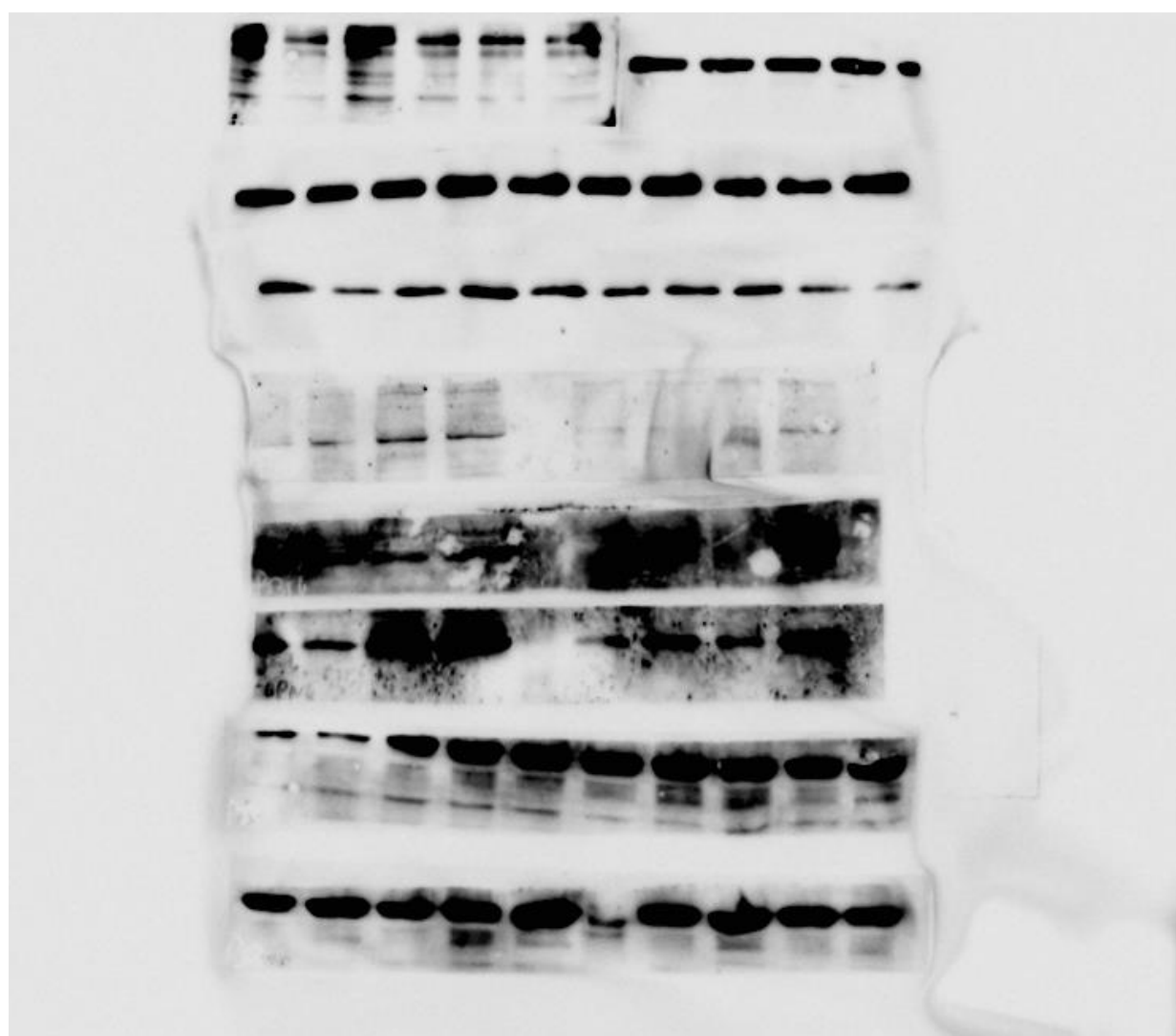

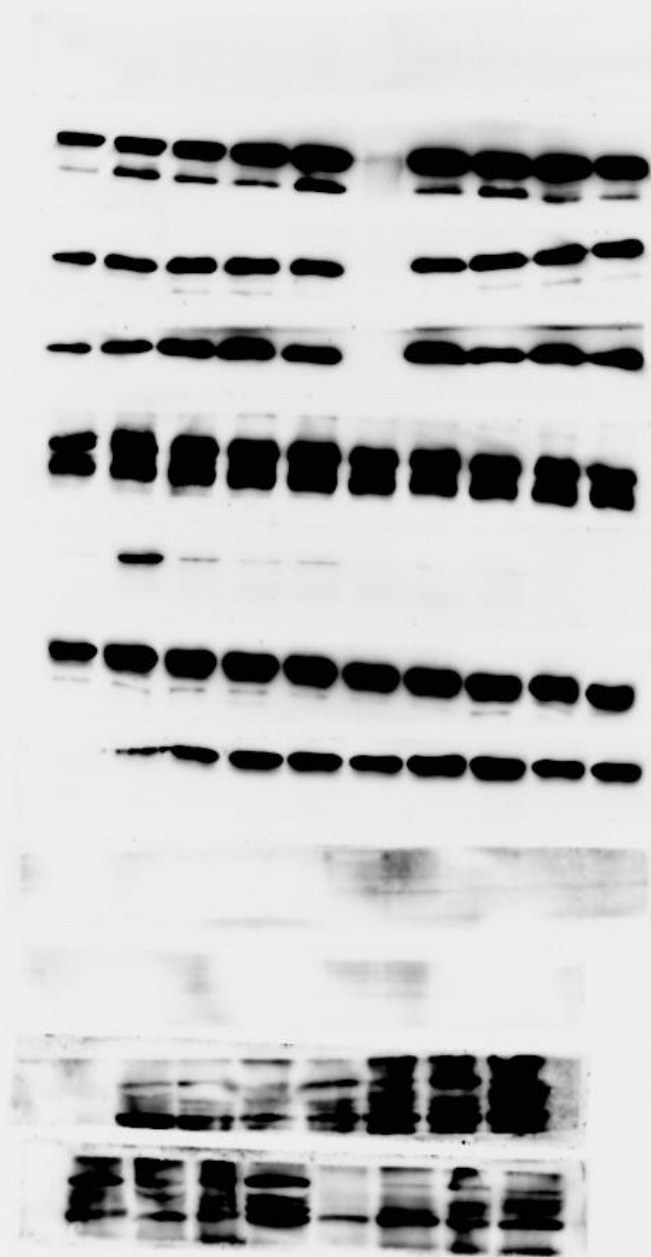

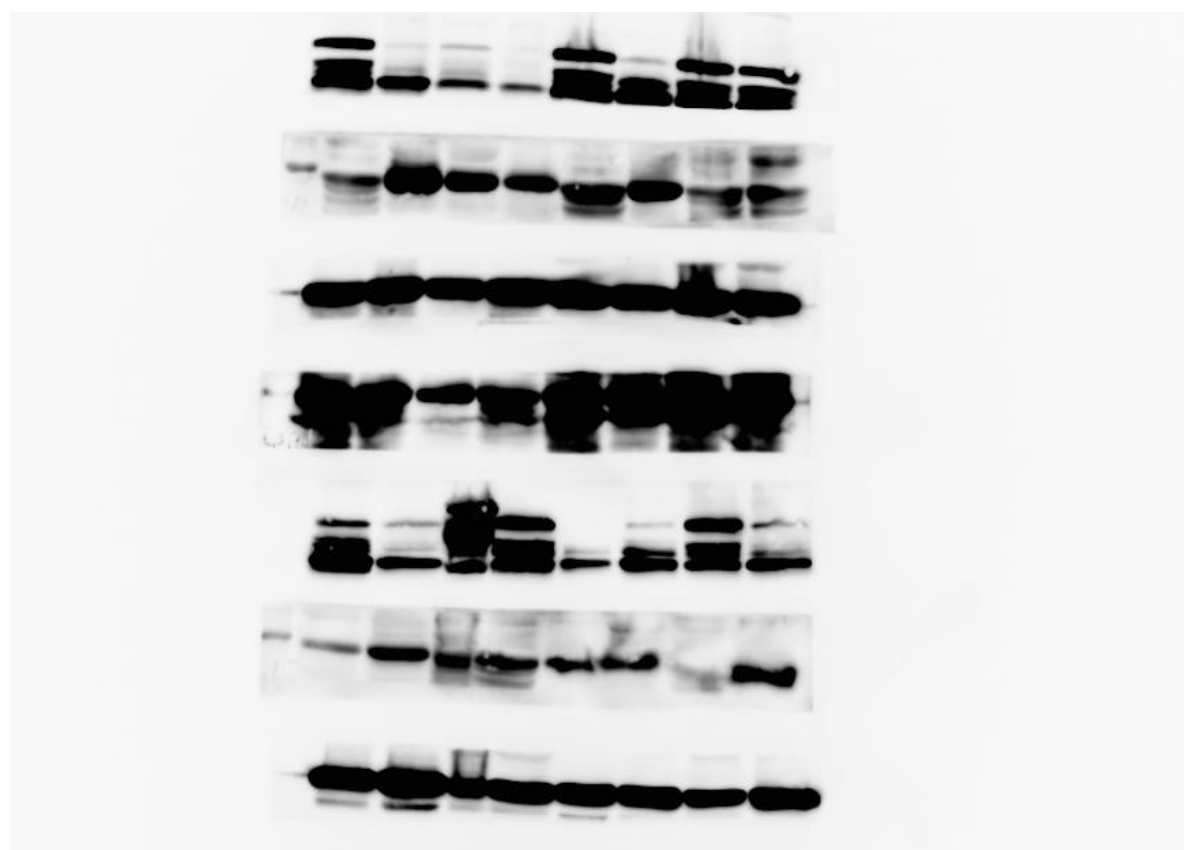

Supplement: Supplementary file 1 — Original Data File [file 41419_2022_4609_MOESM1_ESM.pdf]
